# Exploration of cancer associated fibroblasts phenotypes in the tumor microenvironment of classical and pleomorphic Invasive Lobular Carcinoma

**DOI:** 10.3389/fonc.2023.1281650

**Published:** 2023-12-21

**Authors:** Harsh Batra, Qingqing Ding, Renganayaki Pandurengan, Heladio Ibarguen, Neus Bota Rabassedas, Aysegul Sahin, Ignacio Wistuba, Edwin Roger Parra, Maria Gabriela Raso

**Affiliations:** ^1^ Department of Translational Molecular Pathology, The University of Texas MD Anderson Cancer Center, Houston, TX, United States; ^2^ Department of Pathology, the University of Texas MD Anderson Cancer Center, Houston, TX, United States

**Keywords:** ILC, Invasive Lobular Carcinoma, cancer associate fibroblasts, tumor micro environment (TME), Multiplex immunofluorescence, Computational Pathology

## Abstract

As the second most common subtype of breast carcinoma, Invasive Lobular Carcinoma (ILC) microenvironment features have not been thoroughly explored. ILC has different histological subtypes and elucidating differences in their microenvironments could lead to a comprehensive development of cancer therapies. We designed a custom-made cancer associated fibroblast (CAFs) panel and used multiplex immunofluorescence to identify the differences in tumor microenvironment between Classic ILC and Pleomorphic ILC.

**Materials and methods:**

Multiplex immunofluorescence were performed on formalin fixed paraffin embedded tissues using Opal-7 color kit. The antibodies used for phenotyping CAFs were Pan CK (AE1/AE3), CD45, A-SMA, FAP, S100, Thy-1 with optimized dilutions. The images were acquired and analyzed using Vectra 3.0 imaging system and InForm software respectively.

**Results:**

We studied 19 different CAFs colocalized phenotypes in the tumor, stroma and overall tissue compartments between classic and pleomorphic ILC. Total A-SMA+, A-SMA+FAP+S100+ and A-SMA+S100+ CAFs demonstrated higher densities in classic ILC cases while FAP+S100+ and S-100+ CAFs were increased in the pleomorphic subtype samples.

**Conclusion:**

Our study explores multiple CAFs phenotypes between classical and pleomorphic ILC. We showed that CAFs subset differ between Classic ILC and Pleomorphic ILC. A-SMA CAFs are more prevalent in the TME of classic ILCs whereas Pleomorphic ILCs are dominated by CAFs without A-SMA expression. This also iterates the importance of exploring this particular type of breast carcinoma in more detail, paving the way for meaningful translational research.

## Introduction

The tumor microenvironment (TME) consists of dynamic interactions between extra cellular matrix (ECM), cancer-associated fibroblast (CAFs) and tumor-associated immune cells (TAICs). These components influence and condition tumor metabolism (1), tumor progression (2), and treatment response (3). Recent advances in immunotherapy have led to a growing interest in studying and targeting the tumor microenvironment in order to achieve effective clinical treatments in cancer patients. Notable mentions include examples of immune checkpoint blockade therapies targeting PD-1, PDL-1 interaction (pembrolizumab and nivolumab), CTLA4 blockade (ipilimumab), etc., which aid in tumor evasion from the immune response. In addition, recent studies showcasing immune cell densities, spatial interactions, single-cell profiling results in different tumors, added to the importance of evidence-based translational research. Novel technologies like multiplex immunofluorescence and other high plex tissue profiling assays identify the composition of tumor microenvironment, calculate densities of the different cells present in the tumor microenvironment, identify their topographical distribution and correlates the TME and tumor cells, enabling a comprehensive profiling of the tumor immune landscape. Cornerstone advances, revealing distinct immunologic phenotypes led to the identification of mechanisms of resistance and potential targets to aid in cancer therapy discoveries.

Breast cancer is a leading cause of cancer incidence and deaths worldwide (4). Despite Invasive Lobular Carcinoma (ILC) being the second most common subtype of breast cancer with a distinct biology, most of the studies researching the tumor microenvironment have been done in invasive ductal carcinomas(IDCs) (5–7). ILCs have distinct histological subtypes with the classical subtype being the most common and having a quiescent nature whereas the pleomorphic variant -constituting approximately five percent of total ILC cases-is known to be an aggressive variant (8).

Desmoplasia in tumor tissue is a characteristic feature and stromal fibroblasts and cancer associated fibroblasts (CAFs) are a part of this reaction that form due to the interplay between cancer cells and the surrounding milieu (9–11). While the CAFs resemble normal residing fibroblasts or myofibroblasts, they have a diverse role characterized by increased collagen formation and a greater ECM protein production along with pro-tumor factors that support tumor growth and progression (12–15). Desmoplasia in tumors is caused by different mechanisms like cross-linking of collagens, fiber elongation and realignment (16). CAFs secrete metalloproteinases which are instrumental in desmoplasia and collagen deposition. The interplay of collagen and CAFs might play an intricate role in tumorigenesis especially collagen I, III,V,VI (17). However, it is believed that the turnover or balance of these different collagen types drives the anti-tumorigenicity or pro-tumorigenicity and much remains to be explored regarding the intricacies of interaction of collagen subtypes and CAFs (17).

There are many probable hypothesis about origins of CAF’s, but it is still unclear how they originate (15, 18). Some of the plausible theories include (i) recruitment and activation of resident fibroblasts (19); (ii) epithelial-mesenchymal transition (EMT) of resident epithelial cells (20); (iii) endothelial to mesenchymal transition (EMT) of resident endothelial cells (21) and (iv) differentiation of bone marrow mesenchymal cells (22). A minority of studies have also shown an antitumorigenic capacity of CAFs. For e.g. PD-L1 expression in CAFs of non-small cell lung carcinoma tissues has been demonstrated to correlate with good prognosis, and depletion of CAFs in pancreatic cancer was associated with poor prognosis (23–26).

CAFs heterogeneity is based on phenotypic markers, gene expression profiling, and functionality (11, 27–29). Until now, no single pan-specific CAF marker has been identified and neither a unifying approach to defining, nor a standardized nomenclature for CAF subpopulations has yet emerged. CAF have been shown to express high levels of alpha-smooth muscle actin (α-SMA/*Acta2)* (30)*, PDGFR* α or β (31), CD90 (*Thy1*) (32), fibroblast activation protein (FAP) (33), fibroblast-specific protein 1 (FSP/S100A4) (34), integrin β1/CD29 (28, 35), podoplanin (*Pdpn*) (36), osteonectin (*Sparc*) (37), caveolin 1 (*Cav1*) (35) and vimentin (32). Since CAFs are an essential component of the tumor environment, much interest has arisen to explore their utility as actionable targets in cancer treatment (38).

In the current study, we aimed to profile the CAFs in the tumor microenvironment of breast ILC by comparing two different subtypes (Classic ILC vs Pleomorphic ILC), and explore their spatial distribution concerning the malignant cells using an automated mIF panel image analysis approach.

## Methods

### Sample collection

Twelve primary invasive lobular breast carcinoma surgical specimen whole slide sections, including classic Invasive Lobular Carcinoma (ILC) (n=6) and pleomorphic ILC (n=6) samples, were obtained from departmental archives through IRB protocols approved by Institutional Review Boards at The University of Texas MD Anderson Cancer Center. The retrospective cases selected in our cohort were based on the WHO diagnostic criteria which is based on histology to distinguish the pleomorphic ILC cases and the status of E-Cadherin IHC (negative) to diagnose the case as ILC.

Only the cases which had at least 30% of stromal content by microscopic analysis of hematoxylin and eosin-stained sections by two independent pathologists (MGR and HB) were included in the study. All the cases did not receive neoadjuvant therapy at the time of biopsy. In addition, clinical and pathologic information, including demographic data, age, sex, tumor size, tumor stage and receptor status was collected from medical records. Follow-up information for recurrence-free survival (RFS) and OS rates were also retrieved from the patient’s electronic medical records (**Supplementary Tables 1**, **2**).

### Multiplex immunofluorescence

Formalin fixed paraffin embedded (FFPE) tissue sections were sectioned at 4 μm and stained using antibodies against α-SMA, FSP-1/S-1004A, Thy-1, FAP, CD45 and pan-CK previously validated for immunohistochemistry. 4,6-Diamidino-2-phenylindole (DAPI) was used as counterstain. Each antibody was labeled with a specific fluorophore. Staining was automated (BOND-RX, model B3; Leica Microsystems, Vista, CA, USA). A tyramine signal amplification system-based kit (Opal™ 7-color kit, Akoya/PerkinElmer, Waltham, MA; Cat#NEL797001KT) was used. The primary antibody was detected with horseradish peroxidase (HRP)-conjugated secondary antibody. Once HRP was introduced, the fluorophore tyramide (Amplification Reagent) working solution was added to covalently label the epitope. After the first labeling was complete, the tissue was prepared for detecting the next epitope. This process was repeated automatically. To validate the panel antibodies Positive (colorectal carcinoma tissue) and negative (auto fluorescence) controls were used during each run.

Multiplex immunofluorescence-stained tissues were imaged using the Vectra multispectral imaging system version 3.0 (Akoya Bioscience that measures each fluorescence signal. Multispectral imaging entailed the capturing of an image in low magnification (x10) through the full emission spectrum (10 nm increments between 420 to 720 nm). A trained pathologist selected a region of interest for scanning in high magnification using the Phenochart Software 1.0.9 (931 x 698 µm at 20x resolution). The region of interest selection by a pathologist included areas which had tumor, tumor stroma interface and stroma.

A spectral signature for each fluorophore was obtained using “spectral unmixing library” in the software (InForm™ 2.4.8, Akoya Bioscience) to separate the multispectral image into its individual fluorophores that were then merged into a single image. Algorithms were trained to determine the cellular densities and co-expression of the different markers into 2 compartments, epithelial or stromal, based on the positivity or negativity of CK expression, respectively. The data was consolidated using the software R studio (detailed in the Statistical Analysis section). Finally, the results were expressed as densities of each cell phenotype by mm^2^.

### Exploratory cellular spatial distribution

To calculate each cell population, and for visual display (**Supplementary Table 3**), each cell was classified to one of 18 groups: CK+, CD45+, FAP+, aSMA+, Thy1+, FSP-1+, aSMA+/FAP+, FAP+/Thy1+, FAP+/FSP-1+, aSMA+/Thy1+, aSMA+/FSP-1+, FSP-1+/Thy1+, aSMA+/FAP+/Thy1+, aSMA+/FAP+/FSP-1+, FAP+/FSP-1+/Thy1+, aSMA+/FSP-1+/Thy1+, aSMA+/FAP+/FSP-1+/Thy1+, or “other”. Pericytes are known to express alpha-SMA (39). To avoid misinterpretation, based on histology, we deselected the areas of blood vessels in the stroma and the selected ROIs were composed only of tumor, tumor stroma interface and stroma (excluding the blood vessels), so that pericytes are not included in the final data. We then generated 19 possible marker expression patterns based on the expression of α-SMA, Thy-1, FAP^+^, and FSP-1. In our cohort we had equal numbers of two different subsets of ILC (classic ILC vs Pleomorphic ILC). Patterns of distribution according the nearest neighbor distance were calculated for each CAF phenotype to tumor cells using the X and Y coordinates of each cell with Phenoptr script from R studio (Akoya Biosciences). We then compared the proximities of each CAF subtype to the tumor cells between the two histological subtypes of ILC using IBM SPSS statistics software 24.

### Statistical analysis

For statistical analysis densities and distances were dichotomized in values greater than the median were considered high density or long distance and values equal to or lower than the median was considered low density or close distance. Mann-Whitney and Kruskal-Wallis tests were used to compare phenotype densities between the two tumor subtypes. Two-tailed *p* values <0.05 were considered statistically significant.a

## Results

**Figure 1:**
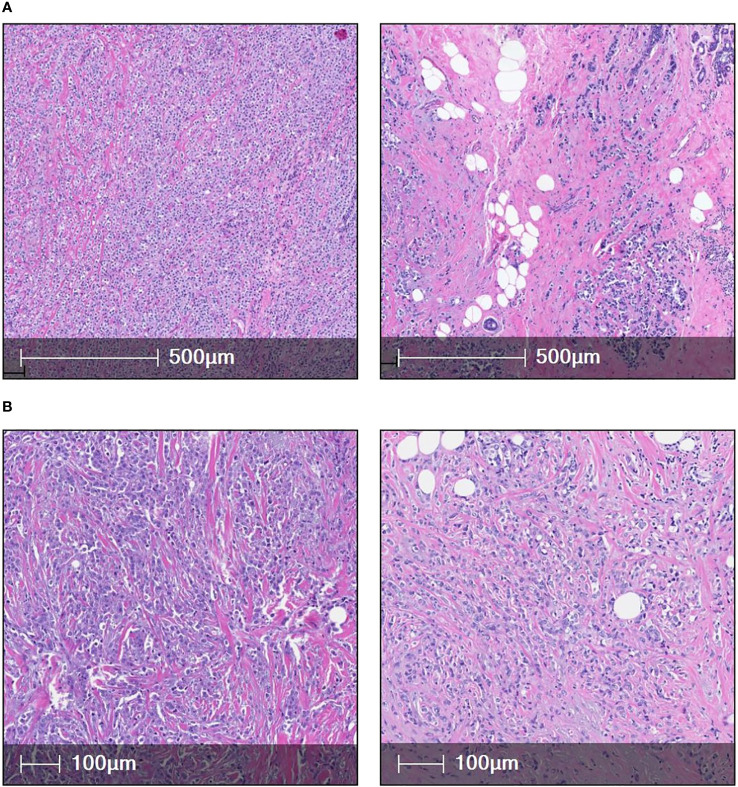
**(A)** [(i) & (ii)] shows two cases of classic ILC(H&E). **(B)** [(iii) & (iv)] shows high magnification images of two cases of pleomorphic ILC (H&E).

Two different histological variants of invasive lobular carcinoma: Classic ILC (n=6) (**Figure 1A**) and Pleomorphic ILC (n=6) (**Figure 1B**) were compared using paraffin embedded (FFPE) samples from individuals diagnosed with non-metastasized classic ILC and pleomorphic ILC we analyzed the tumor microenvironment using multiplex immunofluorescence. Six of these cases were of classic ILC histology and remaining six were of pleomorphic ILC histology. All cases with classical features were nuclear grade 2 and all pleomorphic cases (n=6) were nuclear grade 3. Six were Luminal B, five were Luminal A and one case was triple negative invasive lobular carcinoma with pleomorphic histology.

**Figure 2:**
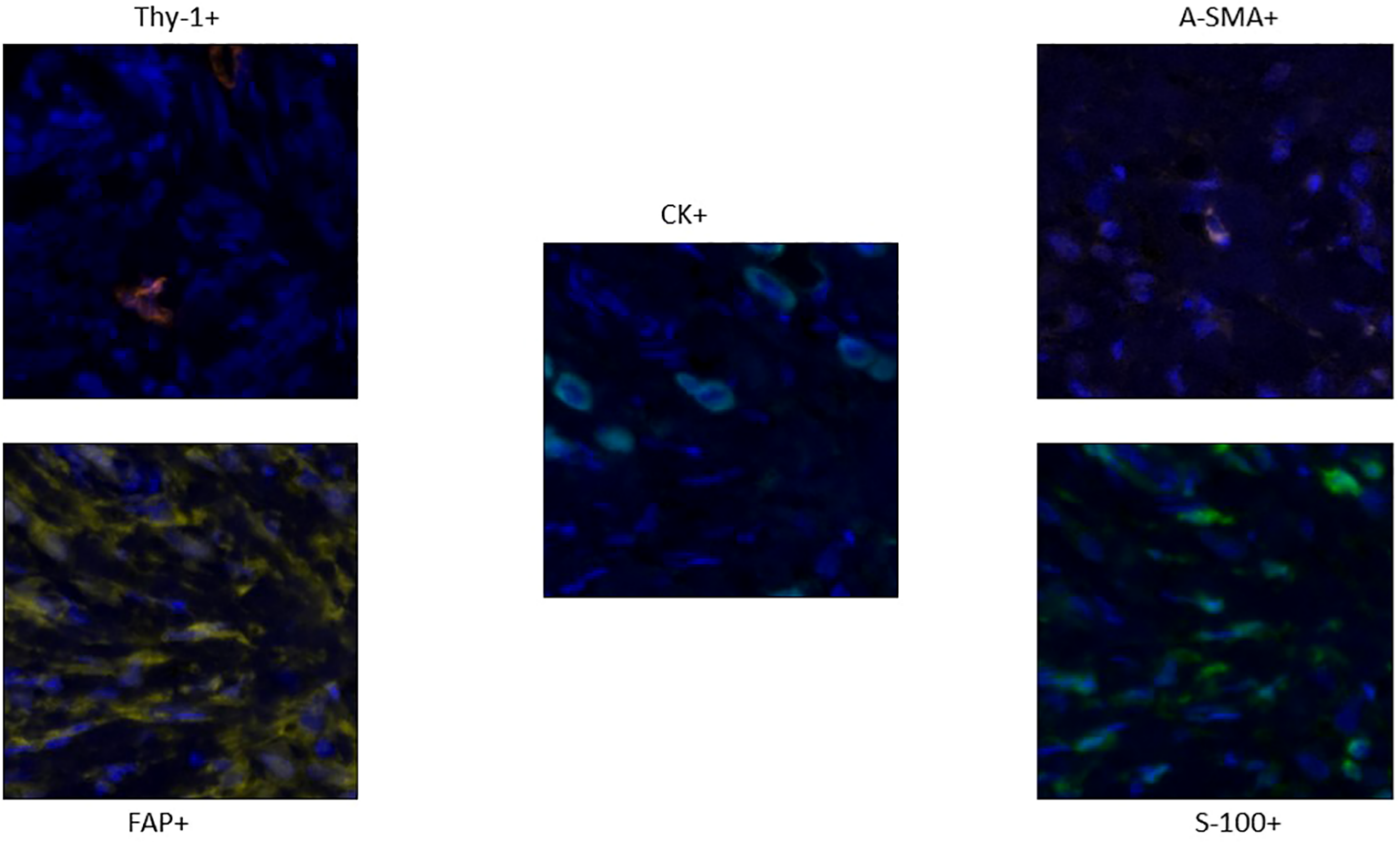
Staining Pattern of different markers in the panel: Thy-1 (orange), FAP+ CAFs (Yellow), A-SMA CAFs (Pink) and S-100 CAFs (Green). The Tumor cells which are CK+ are colored cyan (Centre).

To detect CAF subpopulations, we performed multiplex immunofluorescence to detect markers of hematopoietic cells (CD45), epithelial cells (pan-cytokeratin [CK]), and fibroblasts (α-smooth muscle actin [α-SMA], fibroblast activation protein (FAP), fibroblast specific protein 1 [FSP-1], and Thy-1) on surgically resected invasive lobular carcinoma of the breast (**Figure 2**). We evaluated fibroblasts as cells that are negative for CD45 and CK and positive for at least 1 fibroblast marker (blood vessels were excluded from the region of interest to avoid discrepancies with pericytes positivity for alpha-SMA). Fibroblasts were quantified in epithelial (CK^+^) and stromal (CK^-^) compartments of both diseases. As a proxy for heterogeneity, fibroblasts were classified based on marker expression patterns (single-, double-, triple-, or quadruple-marker^+^) and assigned to 1 of 19 subpopulations based on possible expression patterns of the 4 markers. We observed substantial variation in the subsets of CAFs types and magnitude of infiltration in our breast ILC cohorts, demonstrating the heterogeneity of potential immune responses to the two different tumor subtypes.

**Figure 3:**
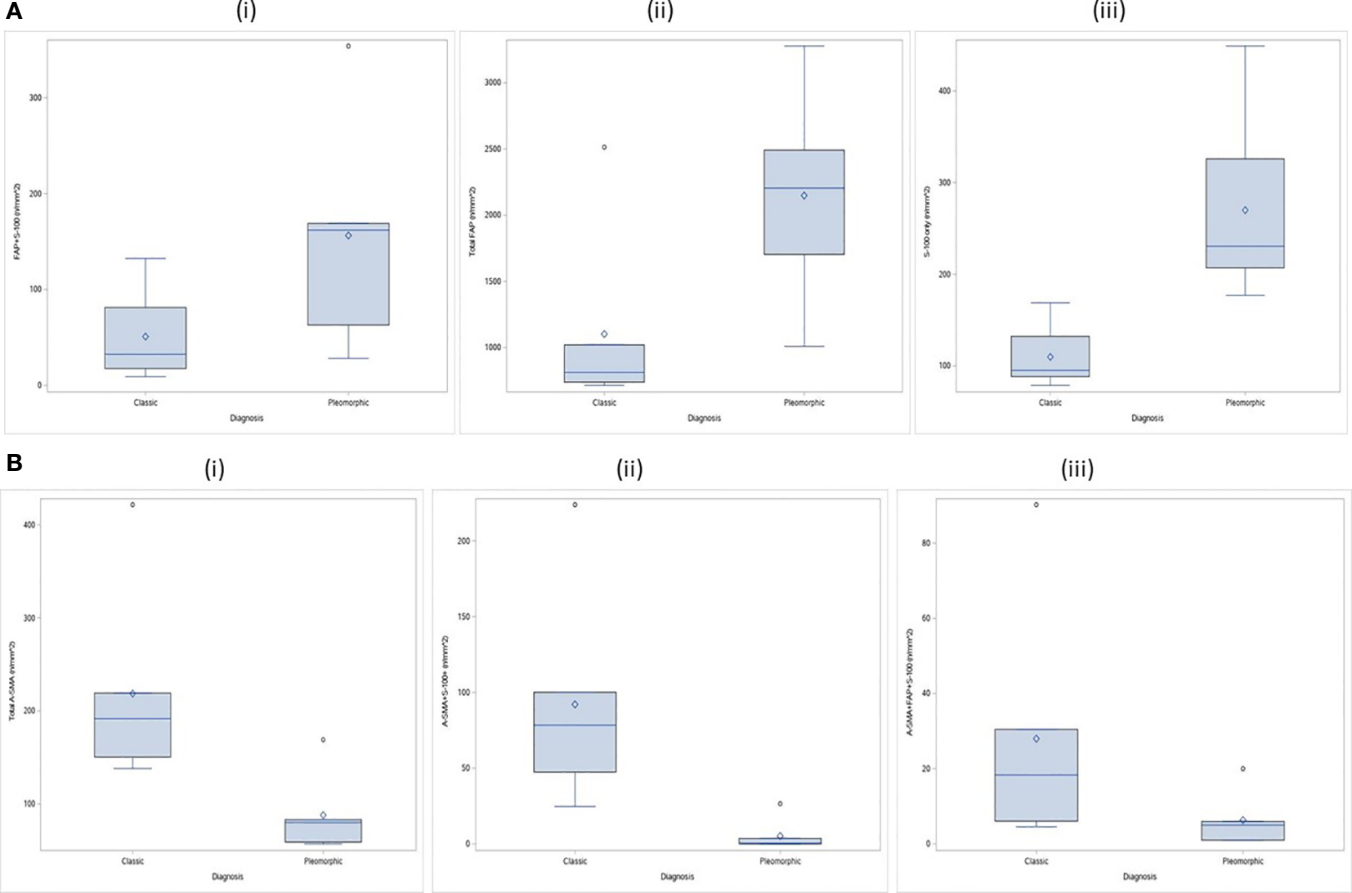
**(A)** CAF Phenotypes which were higher in density (statistically significant) in the TME of pleomorphic ILC as compared to classic ILC: (i) Distribution of FAP+S-100 (n/mm^2) by tumor types in stroma compartment; ii) Distribution of Total FAP (n/mm^2) by tumor types in tumor compartment ; iii) Distribution of S-100 only (n/mm^2) by tumor types in total compartment. **(B)** CAF Phenotypes which were higher in density the TME of classic ILC as compared to pleomorphic ILC: (i) Distribution of Total A-SMA (n/mm^2) by tumor types in total Compartment ; (ii) Distribution of A-SMA+S-100+ (n/mm^2 by tumor types in total Compartment; (iii) Distribution of A-SMA+FAP+S-100 (n/mm^2) by tumor types in total Compartment.

**Figure 4:**
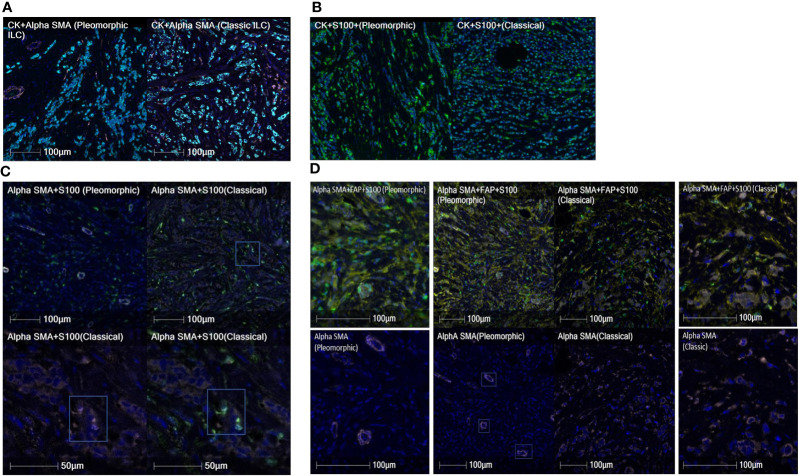
**(A)** Cases showing comparison of A-SMA densities (pink) between pleomorphic ILC (left) and classic ILC (right). The CK+ cells are tumor cells in cyan color. The classic ILC shows a higher density of A-SMA CAFs as compared to pleomorphic ILC. **(B)** Cases showing comparison of S-100 densities (green) between pleomorphic ILC (left) and classic ILC (right). (Tumor cells in cyan). The pleomorphic ILC shows a higher density of S-100 CAFs as compared to classic ILC. **(C)** Cases showing comparison of A-SMA(pink)+S-100(green) co-positive CAF densities between pleomorphic ILC (top-left) and classic ILC (top-right). The CAFs in the classic ILC (box in upper right image) are magnified in the bottom row and shows individual positivity for Alpha SMA (Lower left image) and S100 (lower right image). Note: The A-SMA positive (pink) structures in the pleomorphic ILC are blood vessels and were excluded for data interpretation. **(D)** Cases showing comparison of A-SMA(pink)+FAP(yellow)+S-100(green) copositive CAF densities between pleomorphic ILC and classic ILC (Center panel-Top row). The center panel-bottom row shows absence of Alpha-SMA positivity in CAFs in the pleomorphic ILC case {(the positivity of A-SMA is in the blood vessels (three of them are highlighted in boxes)as examples, not CAFs. The CAFs are either positive for FAP(yellow) or S100 (green) singly or in copositivity}. The side images show high power view of the same for pleomorphic ILC (Left side panel) and classic ILC ( Right side panel).

We found and evaluated nineteen different colocalized phenotypes of CAFs in the tumor, stroma and overall tissue compartments comparing the TME of classic ILC and Pleomorphic ILC. Out of nineteen phenotypes, we found statistically significant differences(p-value<0.05) between classic and pleomorphic ILC in five of the phenotypes, considering overall tissue compartments. The density (cells/mm^2^) of Total A-SMA+, A-SMA+FAP+S100+ and A-SMA+S100 CAFs were higher in classic ILC while densities of FAP+S100+ and S-100+ CAFs were higher in the pleomorphic subtype. In the tumoral compartment, although a statistically significant difference in the density of CAFs was found only in A-SMA+S100+ CAFs (being higher in classic ILC), an “approaching significance” or a trend was seen with Thy-1+, Total FAP+, FAP+ only and FAP+S100+ CAFs being higher in pleomorphic subtype. The results are summarized in **Supplementary Table 3** and **Figures 3A, B**, **4A–D** and **Supplementary Figures 1–4**).

**Figure 5:**
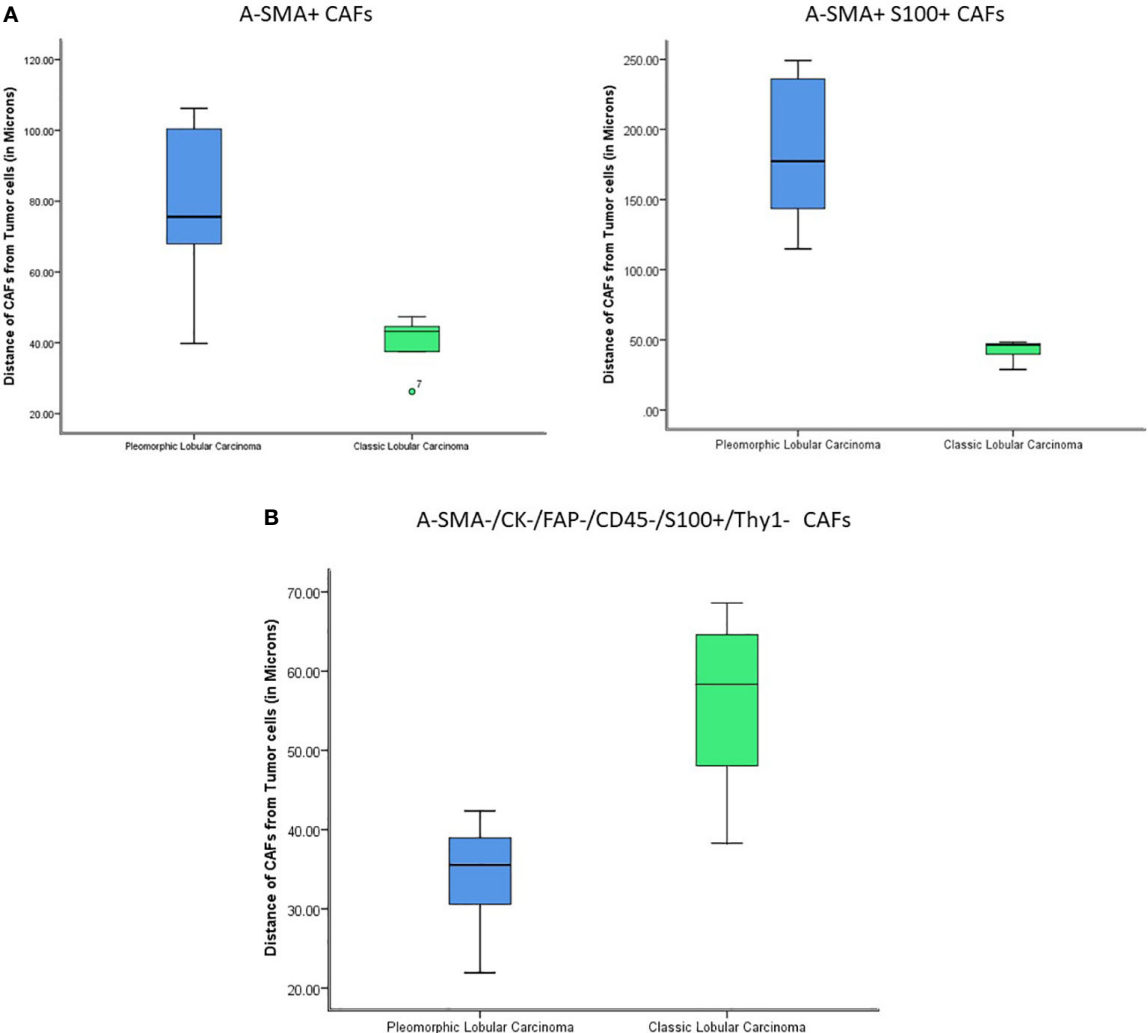
**(A)** The figure shows statistically significant differences in the proximity of CAFs from tumor cells (A-SMA+ and A-SMA+S100+) between Classic ILC and Pleomorphic ILC. (X-axis: tumor type; Y-axis: distance of CAFs from Tumor cells). The A-SMA+ and A-SMA+S100+ CAFs are nearer to tumor cells in Classic ILC as compared to Pleomorphic ILC. **(B)** The figure shows statistically significant differences in the proximity of CAFs from tumor cells (A-SMA-/CK-/FAP-/CD45-/S100+/Thy1-) between Classic ILC and Pleomorphic ILC. (X-axis: tumor type; Y-axis: distance of CAFs from Tumor cells). The A-SMA-/CK-/FAP-/CD45-/S100+/Thy1- CAFs are nearer to tumor cells in Pleomorphic ILC as compared to Classic ILC.

**Figure 6:**
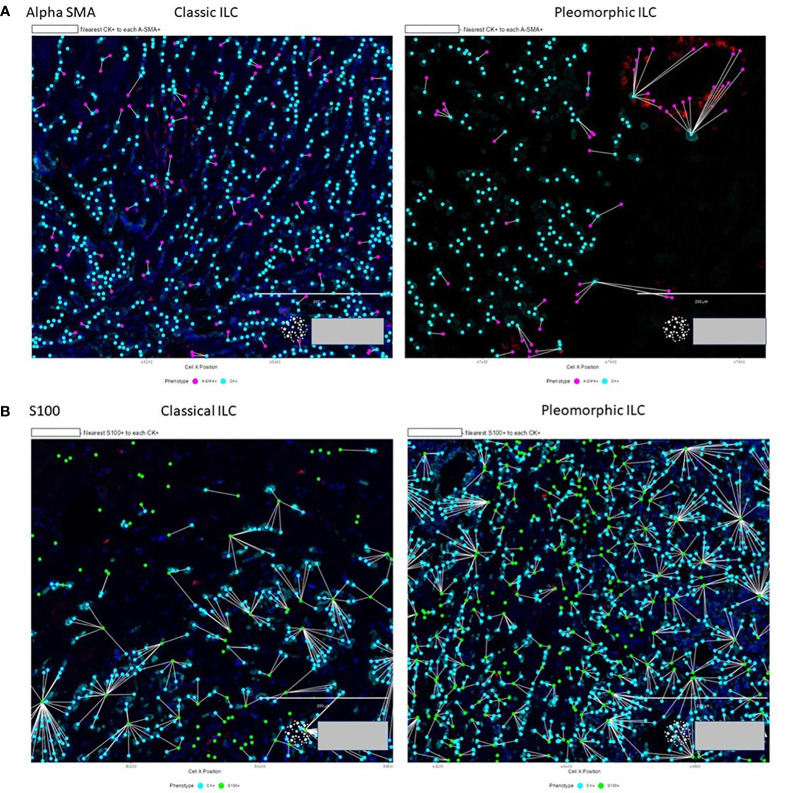
**(A)** Proximity of A-SMA positive CAFs (purple) to tumor cells (cyan) in classic ILC vs pleomorphic ILC. A-SMA+ CAFs are neare to the tumor cells in Classic ILC as compared to Pleomorphic ILC. **(B)** Proximity of S-100 positive CAFs (green) to tumor cells (cyan) in classic ILC (left) vs pleomorphic ILC (right). The S-100+ CAFs are closer to the tumor cells in Pleomorphic ILC as compared to the Classic ILC.

Next, our study aimed to find if there was a difference in the proximity of CAF subtypes to the tumor between the two histologies. Nearest neighbor distances were calculated, and statistically significant differences (p-value<0.05) were observed between the distances of CAFs from the tumor cells in classic and pleomorphic ILC. The A-SMA+, A-SMA+/S100+ phenotypes were closer to the tumor cells in the classic subtype and the FSP1+ only phenotype was closer to the tumor cells in pleomorphic carcinomas. In addition, there were two other phenotypes namely A-SMA+/Thy-1+(closer to tumor cells in classic ILC) and FAP+/S-100+ (closer to tumor cells in pleomorphic ILC), which showed a trend of significance (**Supplementary Table 4** and **Figures 5A, B**, **6A, B**).

We also correlated CAFs differential expression to clinic-pathological variables in each of the tumor subtypes. We did not find any significant correlation of any of the CAF subtype to tumor size, nodal status or Ki67 index.

## Discussion

In this study, we investigated the cancer associated fibroblasts composition in two different subtypes of invasive lobular carcinoma using specially designed multiplex immunofluorescence panels. In this cohort, we had six cases each of classic ILC and Pleomorphic ILC, and we investigated the tumor neighborhood for CAF subtypes and found significant differences in the TME of these two subtypes.

The role of CAF in metastasis is well established, when analyzed as a global population (40) and they serve different functions in tumor biology such as proliferation, metastasis and drug resistance (41–43). CAFs are heterogenous and express different markers either individually or in co-expression and the composition also varies across different tumor types (44, 45)]. CAFs originate from various types of cells, including resident fibroblasts, bone marrow-derived ancestors, or epithelial carcinoma cells that undergo epithelial-mesenchymal transition (17, 46).

CAFs are heterogenous and so care has to be taken in selecting the markers to be used for particular tumor type. For instance, in a study by Ries et al (45) on Pleural mesotheliomas, FAP which is a CAF marker was also shown to be expressed from mesothelioma cells in addition to the fibroblasts. Hence, they propose that FAP is not a good marker to characterize CAFs in Pleural mesotheliomas. Previous studies by Costa et al. and Pelon et al. utilizing flow cytometry have used different CAF markers (SMA, CD29, FAP, FSP1, PDGFRβ, CAV1) profiling them into four different CAF subtypes (CAF-S1 to CAF-S4) across breast and ovarian cancers (CAF-S1: CD29^Med^ FAP^Hi^ FSP1^Low-Hi^ αSMA^Hi^ PDGFRβ^Med-Hi^ CAV1^Low^; CAF-S2: CD29^Low^ FAP^Neg^ FSP1^Neg-Low^ αSMA^Neg^ PDGFRβ^Neg^ CAV1^Neg^; CAF-S3: CD29^Med^ FAP^Neg^ FSP1^Med-Hi^ αSMA^Neg-Low^ PDGFRβ^Med^ CAV1^Neg-Low^; CAF-S4: CD29^Hi^ FAP^Neg^ FSP1^Low-Med^ αSMA^Hi^ PDGFRβ^Low-Med^ CAV1^Neg-Low)^ (28, 47). According to their data, CAF-S1 and CAF-S4 are detected solely in tumors, the CAF-S2 and CAF-S3 subsets are expressed both in tumors and normal tissues. In addition, the studies show that CAF-S1 subtype, which have high Alpha SMA and FAP positivity have clearly demonstrated an immunosuppressive function in breast cancer and attract FOXP3 regulatory T-cells.

Our study which uses multiplex panels also used markers such as A-SMA and FAP used in the studies by Costa et al. and Pelon et al (28, 47), with the difference in two of the markers for CAF identification. Our comprehensive specialized CAF multiplex panel which used protein-based detection consisted of most commonly used markers for CAF: *Alpha-SMA, FAP, S100 and Thy-1 with CD45 and Pan CK* to identify hematopoietic cells and tumor cells respectively. Out of these Alpha-SMA, FAP, FSP1, Thy-1 have been established as CAF markers in other studies (48, 49). A similar multiplex panel to characterize CAFs have been used by Rimm et al (50) in metastatic melanomas with the absence of S100 since they used a 5-plex based panel. We carefully selected these particular markers due to the methodology in use, as the combination of antibodies in multiplex immunofluorescence should be selected with astute care and be best representative of the research question in case. Since our study allowed us for a 7 plex technique we inculcated S100 in our panel in addition to the panel used by Rimm et al (50) to make our panel more comprehensive and be best representative for breast carcinoma Cohort.

Similar to the categorizing technique used by Costa et al. (28), we identified nineteen different phenotypic combinations of CAFs based on colocalization of above markers. This approach helped us to extensively subtype CAFs which can lead to more comprehensive studies at the molecular level in the future. Whereas single cell techniques like cytometry do help in identifying the tumor microenvironment, the power of our study lies in profiling the tumor microenvironment in a better spatial context.

### CAFs in invasive lobular carcinoma

Fibroblast-like cells are the most abundant component in the breast cancer stroma playing a dynamic role in tumorigenesis (44, 51, 52). While most of the work has been done in IDCs, there are only handful of studies exploring the role of CAFs in Invasive lobular carcinoma (53–57). Officially recognized by the WHO in 2003, the Pleomorphic variant of invasive lobular carcinoma constitutes 15% of total ILCs and is considered a more aggressive variant amongst the many variants of ILC (58, 59). Previously described studies have tried to differentiate the microenvironments between IDC and ILCs (53, 60–62). A study by Koo et al. used IHC based study and found that FAP and S100 CAF markers were expressed more in ILC s as compared to IDC(NST). They also compared CAF markers between classic ILC and Pleomorphic ILC in their cohort but found no significant difference. We had a few contrasting results in our study, where we investigated the neighborhood microenvironments between these two dynamically different ILC subtypes, comparing the CAF subpopulation between comparatively quiescent classic ILC with the more aggressive Pleomorphic ILC subtype (63) separately in the tumor compartment, stromal compartment and overall tumor-stroma compartment using multiplex technology.

We found that classic and pleomorphic ILC differed in CAF densities (n/mm^2^) in five of the phenotypes, considering overall tissue compartments. Total A-SMA+, A-SMA+FAP+S100+ and A-SMA+S100+ CAFs were higher in classic ILC while FAP+S100+ and S-100+ CAFs were higher in pleomorphic subtype. We attribute these differences to the technology of multiplexing used in our study which is quantitative in nature compared to semiquantitative and subjective techniques like manual IHC scoring, thus helping in better profiling of the tumor microenvironment.

The role of alpha SMA, FAP and S100 seems to be predominant in the microenvironment of ILCs. ILCs have been shown to have increased alpha SMA CAF population as compared to IDC (NST) in previous studies (56, 64). In a study by Jia et al (65), FAP has been shown to be involved in cell motility and invasion and in another study by Jenkinson et al (65) S-100 has also shown to be involved in cell motility. Our study, comparison of microenvironment of two different histologies of ILC, showed that alpha-SMA+ alone or co-expressed with other pro-tumorigenic CAFs such as FAP or S100 is associated more with the classical ILC as compared to pleomorphic ILCs, which express more FAP positive and S100 positive CAFs (alone or co-expressed) with loss of alpha-SMA expression. These finding suggests microenvironment changes even within the histological subtypes in lobular carcinomas and differences in density of specific CAFs subpopulations could represent a feature of a more aggressive histomorphology. It has been mentioned previously by Reed et al. (66), that Pleomorphic ILC arises along with classic ILC and additional mutations in classic ILC lead to Pleomorphic ILC, however, it remains to be explored whether the specific CAFs phenotypes in the microenvironment originate *de-novo*, separately in the two subtypes or if the mutational changes in Classic ILC also causes the CAFs to acquire changes in phenotypes and be associated with an aggressive histology. These differences in CAF phenotype hold a translational importance of delineating actionable targets in different tumor subtypes, especially in context of CAFs which are heterogenous amongst different tumor types.

Next, our study aimed to find if there was a difference in the proximity of CAFs subtypes to the tumor, when comparing these two histologies. We found that A-SMA+, A-SMA+/S100+ phenotypes were closer to the tumor cells in classic subtype and the S100 only phenotype was closer to the tumor cells in pleomorphic ILC carcinomas. As per our knowledge, ours is the first group to study the proximity of CAFs to the tumor cell in ILCs with similar techniques being employed to see inflammatory cells proximity in mesotheliomas and recently the macrophages in the microenvironment of ILCs (61, 67). These findings also reiterate that the Alpha SMAs have a predominant role in tumor microenvironment of classic ILCs as compared to Pleomorphic variant of ILC. The proximity of a particular phenotype to the tumor presents an important finding as the nearest population of interest (CAFs in this study) interact more intricately with the tumor and targeting these for precision therapy makes for a more plausible approach.

Our study demonstrates that CAFs are heterogenous in nature serving different roles in different tumor microenvironments, demonstrated by the differences in CAFs subpopulations found when comparing in two histopathological varieties of ILC. We demonstrated that different phenotypes of CAFs are localized differentially in ILC subtypes and exhibit specific spatial distribution. This highlights the special biology of ILCs and possibly explains the different microenvironments found in these histological subtypes of ILC. Our small ILC cohort constitutes an exploratory comprehensive analysis of CAFs using protein-based methods and serves as a potential source for future studies investigating the underlying immunobiology of ILCs in a more precise and comprehensive manner.

## Data availability statement

The raw data is confidential and can be obtained after appropriate request to the corresponding author and following appropriate institutional guidelines.

## Ethics statement

The studies involving humans were approved by MDACC Institutional Review Board. The studies were conducted in accordance with the local legislation and institutional requirements. The human samples used in this study were acquired from a by- product of routine care or industry. Written informed consent for participation was not required from the participants or the participants’ legal guardians/next of kin in accordance with the national legislation and institutional requirements. Written informed consent was obtained from the individual(s) for the publication of any potentially identifiable images or data included in this article.

## Author contributions

HB: Conceptualization, Data curation, Formal analysis, Investigation, Methodology, Software, Validation, Visualization, Writing – original draft, Writing – review & editing. QD: Resources, Supervision, Validation, Writing – review & editing. RP: Investigation, Methodology, Software, Writing – review & editing. HI: Methodology, Writing – review & editing. NR: Writing – review & editing. AS: Supervision, Visualization, Writing – review & editing. IW: Funding acquisition, Project administration, Resources, Supervision, Writing – review & editing. EP: Methodology, Supervision, Writing – review & editing. MR: Conceptualization, Funding acquisition, Methodology, Project administration, Resources, Supervision, Validation, Visualization, Writing – original draft, Writing – review & editing.
