# Supplementary material for: Exploration of cancer associated fibroblasts phenotypes in the tumor microenvironment of classical and pleomorphic Invasive Lobular Carcinoma
Source: Front Oncol. 2023 Dec 21;13:1281650. doi: 10.3389/fonc.2023.1281650 (PMC10772146; doi:10.3389/fonc.2023.1281650)
Supplement: Supplementary file 1 [file Table_1.docx]

| S.No | Race | Side | Age at diagnosis(years) | Tumor size(cm) | TNM Staging | ER  (%) | PR  (>1%) | Her2 (Positive if either IHC score 3+ or FISH ratio >2.2) | Ki67 (%) |
| --- | --- | --- | --- | --- | --- | --- | --- | --- | --- |
| Case 1 | \| White \| \| --- \| | Right | 56 | 4 | T2N0M0 | 100 | Positive | P | 50 |
| Case 2 | Black | Left | 51 | 4.2 | T2N1mM0 | 100 | Positive | N | 10 |
| Case 3 | White | Right | 69 | 0.7 | T1BN0M0 | 0 | Negative | N | 20 |
| Case 4 | White | Left | 63 | 4.5 | T2N0M0 | 90 | Negative | P | 30 |
| Case 5 | White | Left | 62 | 4.6 | T2N1AM0 | 90 | Positive | N | 25 |
| Case 6 | Hispanic | Right | 68 | 5.5 | T3N0M0 | 90 | Positive | N | 50 |

Supplementary Table 1: Demographic data of Pleomorphic lobular carcinoma cases

Supplementary Table 2: Demographic data of Classic lobular carcinoma cases

| S.No | Race | Side | Age at diagnosis (years) | Tumor size(cm) | TNM Staging | ER (%) | PR (>1%) | Her2 (Positive if either IHC score 3+ or FISH ratio >2.2) | Ki67  (%) |
| --- | --- | --- | --- | --- | --- | --- | --- | --- | --- |
| Case 1 | \| White \| \| --- \| | Left | 46 | 3.5 | T2N0M0 | 95 | P | N | 5 |
| Case 2 | White | Right | 50 | 2.6 | T2N0M0 | 95 | P | N | NA |
| Case 3 | White | Right | 59 | 3.5 | T2N0M0 | 90 | N | P | 30 |
| Case 4 | White | Right | 80 | 1.5 | T1CN0M0 | 100 | P | N | 5 |
| Case 5 | Hispanic | Left | 80 | 8 | T3N0M0 | 90 | P | N | 10 |
| Case 6 | White | Left | 70 | 1.6 | T2N0M0 | 70 | P | N | 20 |

Supplementary table 3: CAF phenotypes with significant p-values or a trend towards significance highlighted in grey

| S.No | Phenotypes density(n/mm2) | p- value | | |
| --- | --- | --- | --- | --- |
|  |  | Total | Tumor | Stroma |
| 1 | Total_MCs_AE1AE3 | 0.4223 | 0.0927 | 0.6097 |
| 2 | Total_CD45 | 0.255 | 0.5864 | 0.255 |
| 3 | Total_FAP | 0.1093 | 0.0927 | 0.4862 |
| 4 | Total_A_SMA | 0.0104 | 0.255 | 0.0172 |
| 5 | Total__Thy_1 | 0.1495 | 0.0706 | 0.3973 |
| 6 | Total_S_100 | 0.1495 | 0.4862 | 0.3973 |
| 7 | Total__A_SMA_FAP | 0.5211 | 0.3203 | 0.8146 |
| 8 | A_SMA_FAP_Thy_1 | 0.8681 | 0.7585 | 0.9353 |
| 9 | A_SMA_FAP_S_100 | 0.0367 | 0.1704 | 0.2007 |
| 10 | A_SMA_FAP_S_100_Thy_1_ | 0.2947 | 0.2029 | 0.7585 |
| 11 | A_SMA_only | 0.8728 | 0.4855 | 0.9376 |
| 12 | A_SMA_Thy_1 | 0.9375 | 0.7389 | 1 |
| 13 | A_SMA_S_100 | 0.0171 | 0.0222 | 0.0172 |
| 14 | A_SMA_S_100_Thy_1 | 0.317 | 0.2029 | 0.2518 |
| 15 | FAP_only | 0.6966 | 0.0927 | 0.9376 |
| 16 | FAP_S_100 | 0.0374 | 0.0927 | 0.0927 |
| 17 | FAP_S_100_Thy_1 | 0.5722 | 0.2029 | 0.4706 |
| 18 | FAP_Thy_1 | 0.9376 | 0.1208 | 0.6966 |
| 19 | S_100_only | 0.0172 | 0.3973 | 0.0305 |
| 20 | S_100_Thy_1 | 0.3957 | 0.9353 | 0.3973 |
| 21 | Thy_1_only | 0.3965 | 0.1564 | 0.3973 |
| 22 | CK_A_SMA | 0.9376 | 0.8146 | 1 |

Supplementary Table 4: CAF phenotypes with significant p-values or a trend towards significance highlighted in grey.

| S.No | Distance of CK+ cells from CAF subtype | p-value |
| --- | --- | --- |
| 1 | Total FAP | 0.093 |
| 2 | Total Alpha-SMA | 0.026 |
| 3 | Total Thy1 | 0.132 |
| 4 | Total S100 | 0.31 |
| 5 | Alpha-SMA/FAP | 0.132 |
| 6 | Alpha-SMA/FAP/Thy1 | 0.4 |
| 7 | Alpha-SMA/FAP/S100 | 0.093 |
| 8 | Alpha-SMA/FAP/S100/Thy1 | 1 |
| 9 | Alpha SMA only | 0.699 |
| 10 | Alpha-SMA/Thy1 | 0.067 |
| 11 | Alpha-SMA/S100 | 0.02 |
| 12 | Alpha-SMA/S100/Thy1 | 1 |
| 13 | FAP only | 0.31 |
| 14 | FAP/S100 | 0.065 |
| 15 | FAP/S100/Thy1 | 0.229 |
| 16 | FAP/Thy1 | 0.485 |
| 17 | S100 only | 0.009 |
| 18 | S100/Thy1 | 0.792 |
| 19 | Thy1 only | 0.394 |
