# Supplementary material for: Exploration of cancer associated fibroblasts phenotypes in the tumor microenvironment of classical and pleomorphic Invasive Lobular Carcinoma
Source: Front Oncol. 2023 Dec 21;13:1281650. doi: 10.3389/fonc.2023.1281650 (PMC10772146; doi:10.3389/fonc.2023.1281650)
Supplement: Supplementary file 2 [file Image_1.pdf]

Supplementary figures

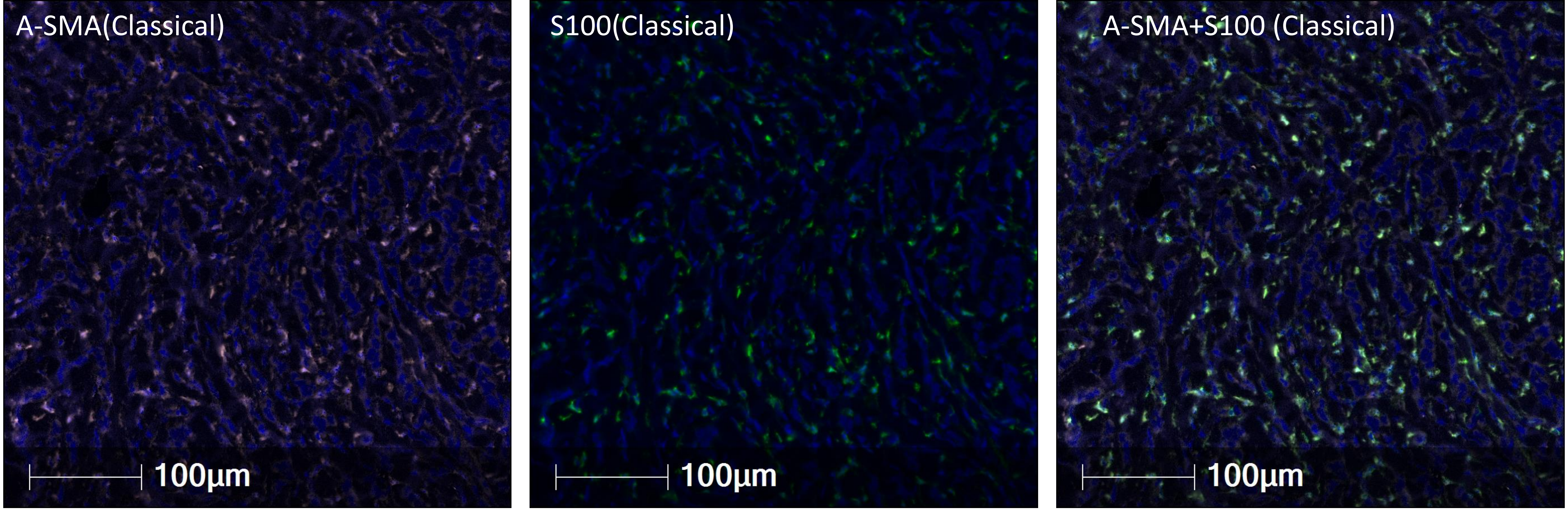

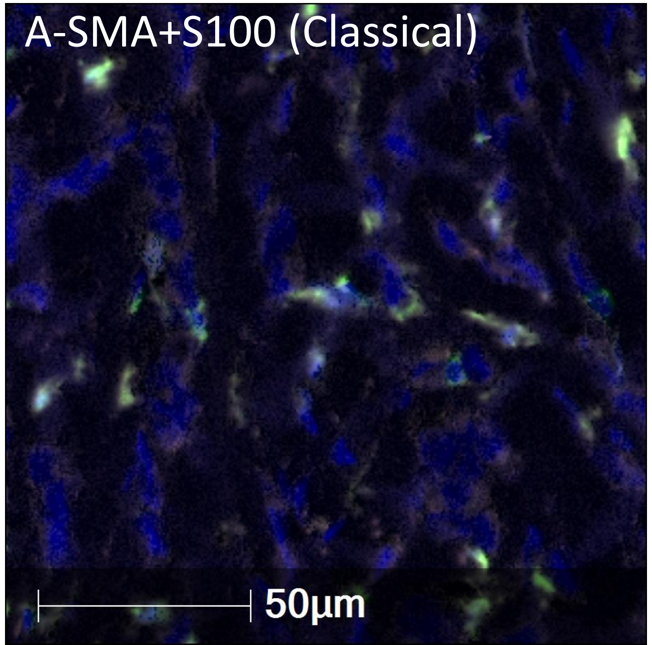

Supplementary figure 1 : Cases showing A-SMA(pink)+S-100(green) co-positive CAF densities in classic ILC. The CAFs in the classic ILC are magnified in the bottom image and shows co-positivity for Alpha SMA and S100.

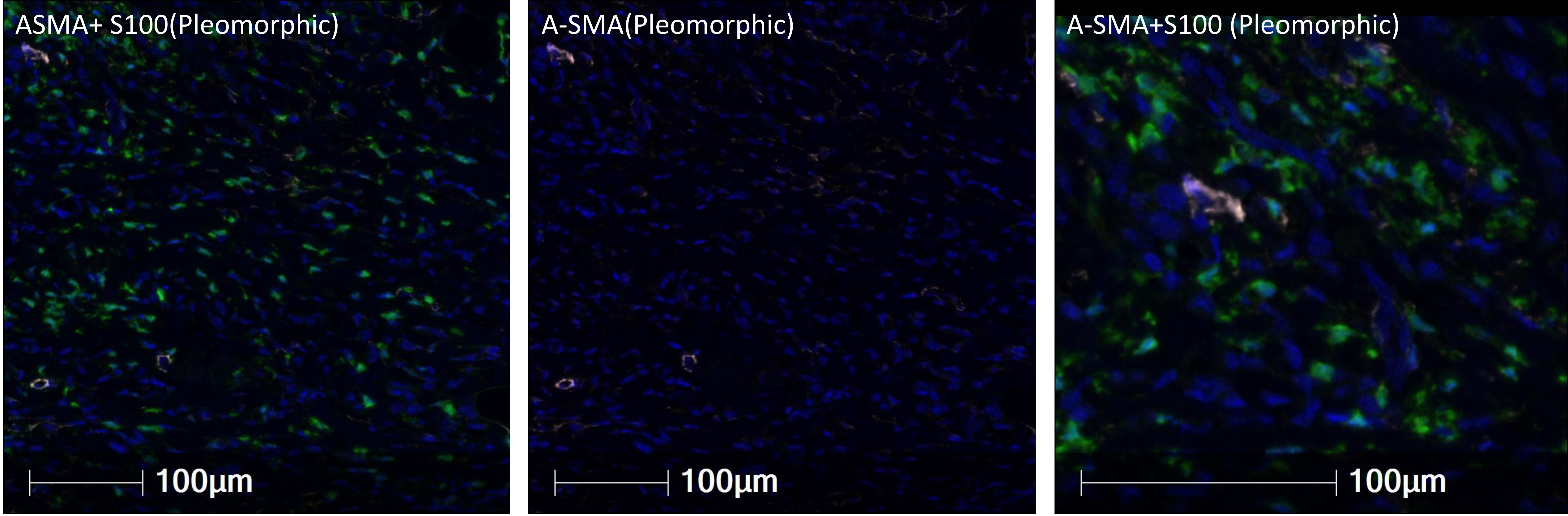

Supplementary figure 2 : Cases showing A-SMA(pink)+S-100(green) co-positive CAF densities in pleomorphic ILC (left) . Center image shows individual positivity for Alpha SMA.

High power view of A-SMA(pink)+S-100(green) CAFs in Pleomorphic ILC (extreme-right)

Note: The A-SMA positive (pink) structures in the pleomorphic ILC are blood vessels and were excluded for data interpretation. The CAFs in pleomorphic ILC are very scarcely copositive for both markers, compared to Classic ILC where the density of A-SMA+S100 copositive CAFs are higher in density (refer Fig. 4C)

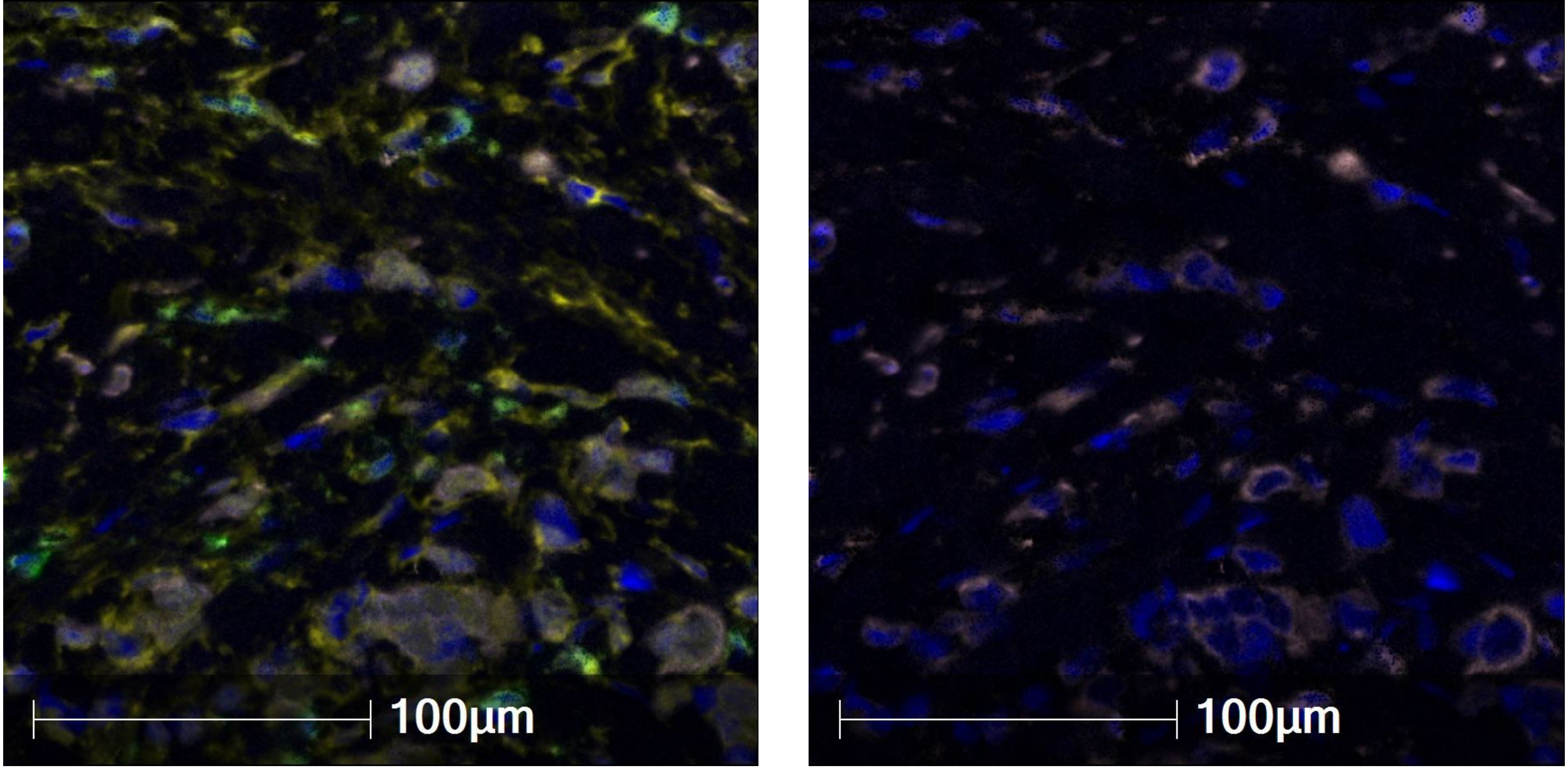

Supplementary figure 3 : High power view of A-SMA+FAP+S100 CAFs of the same case (Fig 4D) of Classic ILC. The density of copositive A-SMA+FAP+S100 CAFs in Classic ILC is higher than Pleomorphic ILC.

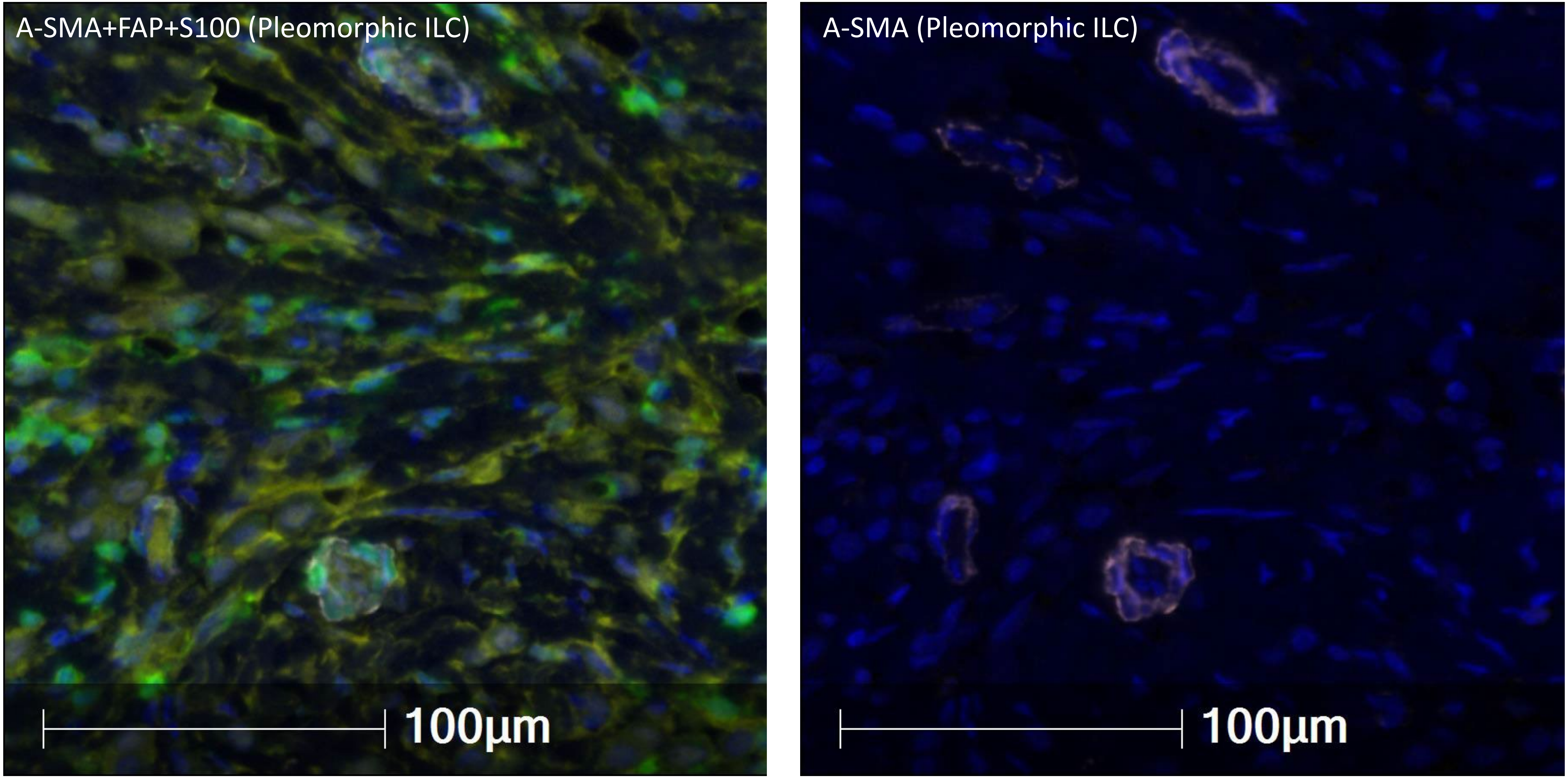

Supplementary figure 4 : High power view of A-SMA+FAP+S100 CAFs of the same case (Fig 4D) of pleomorphic ILC. The CAFs are negative for A-SMA (Only blood vessels are positive). The CAFs are FAP+S100 copositive. The density of copositive A-SMA+FAP+S100 CAFs in Pleomorphic ILC is less than Classic ILC
